# Maxillary Sinus Papillary Edema as a Predictor of Odontogenic Sinusitis

**DOI:** 10.1002/lary.70323

**Published:** 2025-12-19

**Authors:** Hussein Mackie, Japnam Jassal, Carl Wilson, Wamidh Alkhoory, Jacob G. Eide, Edward D. McCoul, John R. Craig

**Affiliations:** ^1^ Michigan State University College of Human Medicine East Lansing Michigan USA; ^2^ Department of Otolaryngology—Head and Neck Surgery Henry Ford Health Detroit Michigan USA; ^3^ Department of Public Health Sciences Henry Ford Health Detroit Michigan USA; ^4^ Henry Ford Health Department of Pathology Detroit Michigan USA; ^5^ Department of Otolaryngology—Head and Neck Surgery Mayo Clinic Rochester Minnesota USA; ^6^ Department of Otorhinolaryngology Ochsner Health New Orleans Louisiana USA; ^7^ Department of Otolaryngology—Head and Neck Surgery Michigan State University College of Human Medicine Lansing Michigan USA

**Keywords:** apical periodontitis, chronic rhinosinusitis, endoscopic sinus surgery, nasal polyps, odontogenic sinusitis, oroantral fistula

## Abstract

**Objectives:**

Odontogenic sinusitis (ODS) refers to purulent sinusitis caused by infectious maxillary dental pathologies, with > 90% occurring unilaterally. While various clinical features suggest ODS, diagnosing some ODS patients can be challenging. This study aimed to determine the value of maxillary sinus papillary edema (MSPE) in predicting ODS.

**Methods:**

A retrospective cohort study was conducted on patients who underwent at least maxillary antrostomy for unilateral sinus disease (USD). Adequately powered samples of three USD cohorts were generated: ODS, infectious maxillary CRS without nasal polyps (CRSsNP), and noninfectious CRSwNP. Multiple variables were collected, but the primary outcome was MSPE presence via double‐blinded endoscopic image review by three rhinologists. Inter‐rater agreement was calculated, and a predictive model for MSPE was generated.

**Results:**

Of 41 ODS, 22 infectious CRSsNP, and 13 noninfectious CRSwNP patients, mean age was 57.0 years and 55.3% were female. There was almost perfect agreement between rhinologists when assessing for MSPE on endoscopic images (*κ* = 0.927). MSPE was seen in 100% of ODS, 22.7% infectious CRSsNP, and 0% CRSwNP. MSPE was significantly more likely in ODS compared to non‐odontogenic CRS (*p* < 0.0001), demonstrating 100% sensitivity and 89.1% specificity for ODS, with 85.7% positive and 100% negative predictive values.

**Conclusions:**

MSPE is a distinct finding that is reliably identifiable and is significantly more likely in ODS compared to infectious CRSsNP and noninfectious CRSwNP. With 100% sensitivity and NPV, MSPE absence makes ODS very unlikely. With 86% PPV, MSPE is not pathognomonic for ODS, but should arouse suspicion for a possible odontogenic source.

**Level of Evidence:**

3.

## Graphical abstract

Maxillary sinus papillary edema (MSPE) is a distinct finding that is reliably identifiable, and is significantly more likely in odontogenic sinusitis (ODS) compared to infectious chronic rhinosinusitis without nasal polyps and noninfectious chronic rhinosinusitis with nasal polyps. With 100% sensitivity and NPV, MSPE absence makes ODS very unlikely. With 86% PPV, MSPE is not pathognomonic for ODS, but should arouse suspicion for a possible odontogenic source.

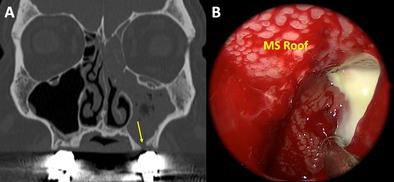

## 1 Introduction

Odontogenic sinusitis (ODS) refers to bacterial sinusitis caused by infectious maxillary dental pathologies or complications from dental procedures [1]. ODS presents unilaterally in > 90% of cases [2], and is the most common cause of unilateral maxillary sinus opacification on computed tomography (CT) [3, 4, 5]. Importantly, ODS is also a very common cause of infectious extra‐sinus complications [6, 7]. Improving ODS recognition will not only improve treatment planning for uncomplicated ODS, it may help prevent potentially vision‐ and life‐threatening complications in complicated ODS as well [8].

Diagnosing ODS involves confirming infectious maxillary sinusitis with nasal endoscopy and CT, and adjacent infectious maxillary dental pathology with appropriate dental evaluation and imaging [1]. Diagnosing ODS can prove challenging since patients present with symptoms that are often indistinguishable from non‐odontogenic rhinosinusitis, and since they rarely report dental symptoms, odontogenic sources may be overlooked. Clinical features like unilateral maxillary sinus or middle meatal purulence, foul smell, and odontogenic bacteria identified on sinus culture increase the likelihood of ODS [9, 10], but the diagnosis can still be elusive. Complicating diagnosis further, up to one‐third of ODS patients demonstrate no overt dental pathology on CT [9, 11]. In such situations, clinicians must try to suspect ODS based on the aforementioned clinical features, but none is truly pathognomonic for ODS. While the majority of ODS is unilateral, the differential diagnosis of unilateral sinus disease (USD) is broad [3]. Differentiating the different forms of USD is critical toward providing patients the most definitive medical and surgical care.

Nasal endoscopic findings have received less attention than CT findings when evaluating different types of sinus disease. However, as endoscopic visual quality has improved over time, there are nuances to endoscopic viewing that can prove helpful diagnostically and therapeutically. For example, McCoul et al. proposed multiple practical suggestions when evaluating patients endoscopically, with one being to assess the mucosal surface for subtle inflammatory changes [12]. Interestingly, ODS patients often demonstrate a characteristic appearance on the surface of their edematous mucosa, originally described by Zhang et al. as fine papillary mucosal folds ODS [13]. While maxillary sinus papillary edema (MSPE) has been reported to be characteristic of ODS [13, 14], its presence has not been compared between patients with ODS versus non‐odontogenic chronic rhinosinusitis (CRS). The purpose of this study was to determine the diagnostic value of MSPE in predicting ODS.

## 2 Methods

A retrospective cohort study was conducted on consecutive adult patients who underwent at least maxillary antrostomy during endoscopic sinus surgery (ESS) for different types of USD, from December 2017 until achieving adequate sample sizes for each cohort. The Henry Ford Health Institutional Review Board approved this study. An a priori power analysis was conducted to determine sample sizes for three different USD groups (80% power): ODS, non‐odontogenic infectious maxillary CRS without nasal polyps (CRSsNP), and noninfectious CRS with nasal polyps (CRSwNP). Based on this analysis, 41 ODS, 22 infectious CRSsNP, and 13 noninfectious CRSwNP patients were required to power the study adequately.

ODS was defined by patients having at least middle meatal purulence adjacent to confirmed maxillary dental pathology [1]. Maxillary dental pathology was always confirmed by dental specialists by examination and dental imaging. Apical periodontitis (endodontic infection) was confirmed by pulp vitality testing showing pulpal necrosis, and cone‐beam CT imaging to detect periapical lesions. Patients were diagnosed as ODS after extraction (without oroantral fistula) by a combination of the dental specialist detecting an oroantral communication at time of extraction, symptoms developing after the extraction, and maxillary sinus cultures demonstrating likely oral bacteria. For ODS with oroantral fistula, a fistula was determined by visualization or probing. For ODS in the setting of maxillary bone augmentations and/or dental implants, implant surgeons determined by whether they were treatable infectious sources. Of note, all ODS patients in this study had overt dental pathology on CT in addition to being confirmed by dental specialists. Infectious CRSsNP was defined as nasal endoscopic evidence of maxillary sinus purulence in patients with no confirmed maxillary dental pathology. If patients were suspected preoperatively to have CRSsNP based on sinus CT showing no overt dental pathology, but oral organisms were identified on sinus cultures after ESS, they were referred to an endodontist for endodontic evaluation. All CRSwNP patients had unilateral inflammatory polyps with no sinonasal purulence. Patients were excluded if they received oral antibiotics or steroids in the 4 weeks leading up to surgery, and if they had autoimmune disorders or primary or acquire immunodeficiencies.

Clinical data collected included patient demographics (age, gender, ethnicity), sinus pathologies, dental pathologies in the ODS cohort, side of disease, primary or revision ESS, presence of tissue eosinophilia (≥ 10 eosinophils/high‐powered field) [15], duration of sinusitis symptoms (months), preoperative 22‐item sinonasal outcome test (SNOT‐22), presence of maxillary sinus edema and MSPE, and preoperative CT grading of the maxillary, anterior and posterior ethmoid, sphenoid, and frontal sinuses (graded as none/mucosal thickening or partial to complete opacification). Microbiological analyses were also performed in patients with ODS and infectious CRSsNP. Purulence was collected in sterile fashion from maxillary sinuses into mucus specimen traps (Covidien, Mansfield, MA). Specimens were immediately submitted to the core microbiology laboratory for routine aerobic and anaerobic culture and identification [10]. Select formalin‐fixed tissue sections that were stained with hematoxylin and eosin (H&E) were also selected from each patient cohort to highlight their histologic features.

The primary outcome measure was MSPE presence on endoscopic images, and this was determined via double‐blinded review of surgical video recordings. First, one author (H.M.), blinded to pathologies, reviewed endoscopic recordings of each included case and saved 70° still digital images of the maxillary sinus after completed maxillary sinus surgery. Still images were de‐identified and randomized for blinded review by the senior author and two rhinologists from other institutions (J.G.E., E.D.M.). First, each surgeon was trained on 5 representative endoscopic still images of MSPE, as well as 5 examples of mucosal edema without papillary changes. None of the training images were from the test dataset. The surgeons then reviewed the study patients' images to assess for maxillary sinus edema and MSPE, and inter‐rater agreement was calculated. Discrepancies were reviewed and consensus was reached on MSPE presence for purposes of subsequent statistical analyses.

Statistical analysis was performed using SAS v9.4 (SAS Institute Inc., Cary, NC). Numerical values were described as medians and interquartile ranges (Q1—Q3) and were compared via Kruskal–Wallis test. If a significant *p* value was observed from the Kruskal–Wallis test on 3 × 2 comparisons, then post hoc pairwise comparisons were conducted to determine which groups were significantly different via the Dwass, Steel, Critchlow‐Fligner method, which also corrected for multiplicity. Categorical variables were described via frequencies and percentages and were compared between different groups via *χ* ^2^ test of independence, or Fisher's exact test if sparse data was present (counts < 5). Inter‐rater agreement between the three rhinologists' endoscopic assessments for MSPE was analyzed with the Fleiss *κ* test. Models were created first for MSPE in predicting ODS compared to all CRS patients. Sensitivity, specificity, positive predictive value (PPV), and negative predictive value (NPV) were calculated. For microbiological analyses, rates of individual bacteria as well as groups of oral and anaerobic were compared between ODS and infectious CRSsNP cohorts.

## 3 Results

**FIGURE 1:**
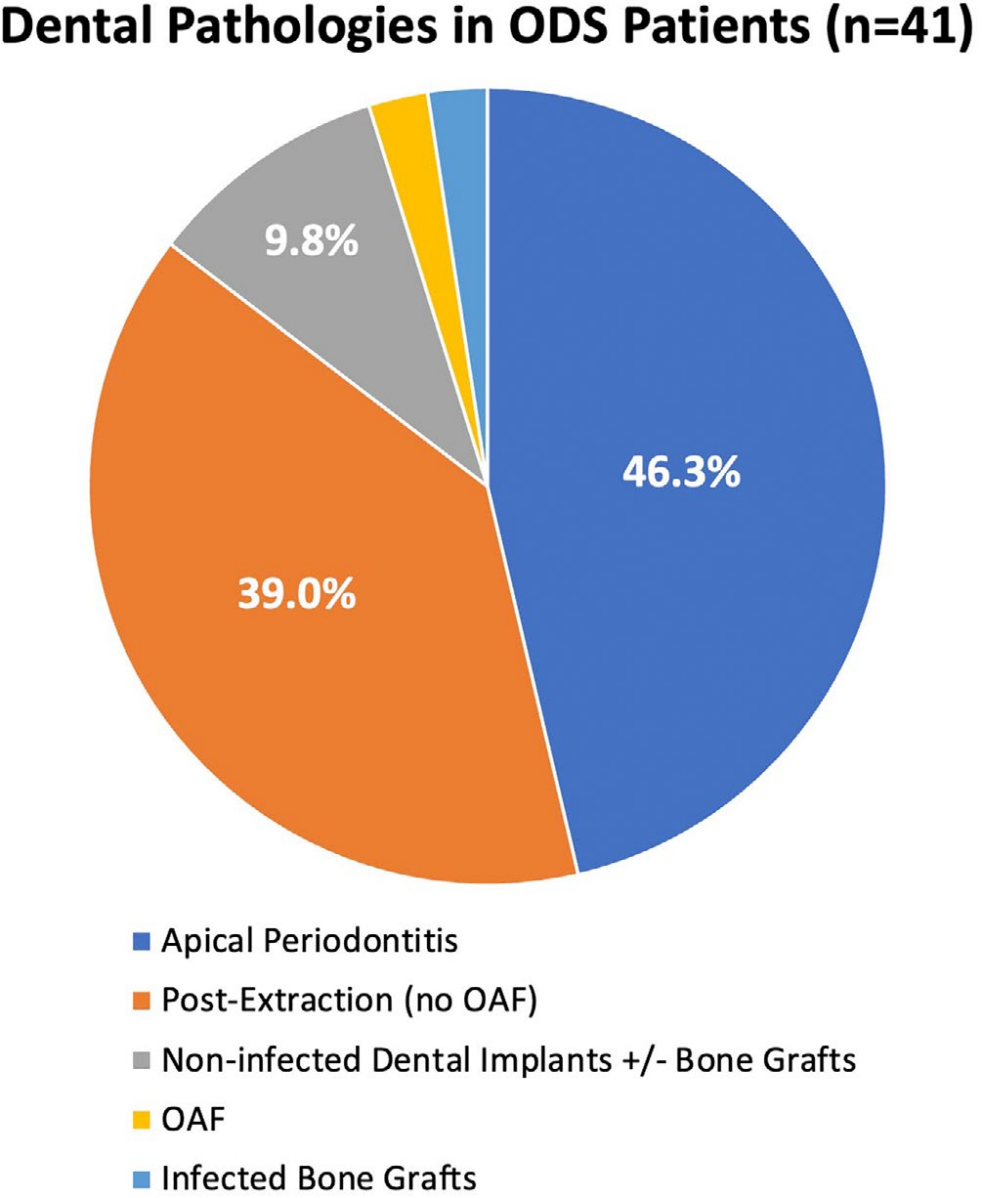
Frequencies of different causative dental pathologies in the odontogenic sinusitis (ODS) cohort. OAF, oroantral fistula.

Table 1 shows demographic and clinical data compared across all three patient cohorts before adjusting for multiplicity. After pairwise comparisons and adjusting for multiplicity (not shown in tables), ODS and infectious CRSsNP patients demonstrated no significant differences in any of the Table 1 variables. There were significantly more females with ODS compared to noninfectious CRSwNP patients (63.4% vs. 23.1%, *p* = 0.032). There were also higher rates of tissue eosinophilia in CRSwNP compared to infectious CRSsNP (69.2% vs. 26.3%, *p* = 0.047), but not to ODS (*p* = 0.104). Additionally, revision ESS was significantly more likely in infectious CRSsNP (63.6%) compared to both ODS (0.0%) and CRSwNP (0.0%; *p* = 0.0002 for each cohort comparison). Figure 1 shows the rates of different dental pathologies causing ODS in the ODS group.

**TABLE 1:** Demographic and clinical data for the three cohorts with unilateral sinus disease: Odontogenic sinusitis (ODS), infectious chronic rhinosinusitis without nasal polyps (CRSsNP), and noninfectious chronic rhinosinusitis with nasal polyps (CRSwNP).

	ODS (*n* = 41)	Infectious CRSsNP (*n* = 22)	Noninfectious CRSwNP (*n* = 13)	*p*
Age [years; median (IQR)]	58.0 (50.0, 67.0)	60.5 (49.0, 72.0)	59.0 (47.0, 66.0)	0.687
Gender [*n*, (%)]				
Female	26 (63.4)	13 (59.1)	3 (23.1)	**0.035**
Male	15 (36.6)	9 (40.9)	10 (76.9)	
Ethnicity [*n*, (%)]				
White	31 (75.6)	13 (59.1)	8 (66.7)	0.164
Black	9 (22.0)	5 (22.7)	3 (25.0)	
Indian	0 (0.0)	2 (9.1)	0 (0.0)	
Hispanic	0 (0.0)	2 (9.1)	0 (0.0)	
Arab	1 (2.4)	0 (0.0)	1 (8.3)	
Sidedness [*n*, (%)]				
Left	20 (48.8)	13 (59.1)	4 (30.8)	0.2693
Right	21 (51.2)	9 (40.9)	9 (69.2)	
Eosinophilic [*n*, (%)]	13 (36.1)	5 (26.3)	9 (69.2)	**0.042**
Symptom duration (months), [median (IQR)]	5.0 (2.0, 12.0)	12.0 (3.0, 36.0)	12.0 (6.0, 24.0)	0.131
SNOT‐22 [median (IQR)]	28.5 (15.5, 47.0)	20.5 (12.5, 50.5)	42.0 (31.0, 53.0)	0.225
Revision ESS [*n*, (%)]	0 (0.0)	14 (63.6)	0 (0.0)	**< 0.0001**

*Note*: Bolded *p* values were statistically significant. Bold values show statistically significant *p* values prior to pairwise comparisons.

Abbreviations: ESS, endoscopic sinus surgery; IQR, interquartile range; SNOT‐22, 22‐item sinonasal outcome test.

**FIGURE 2:**
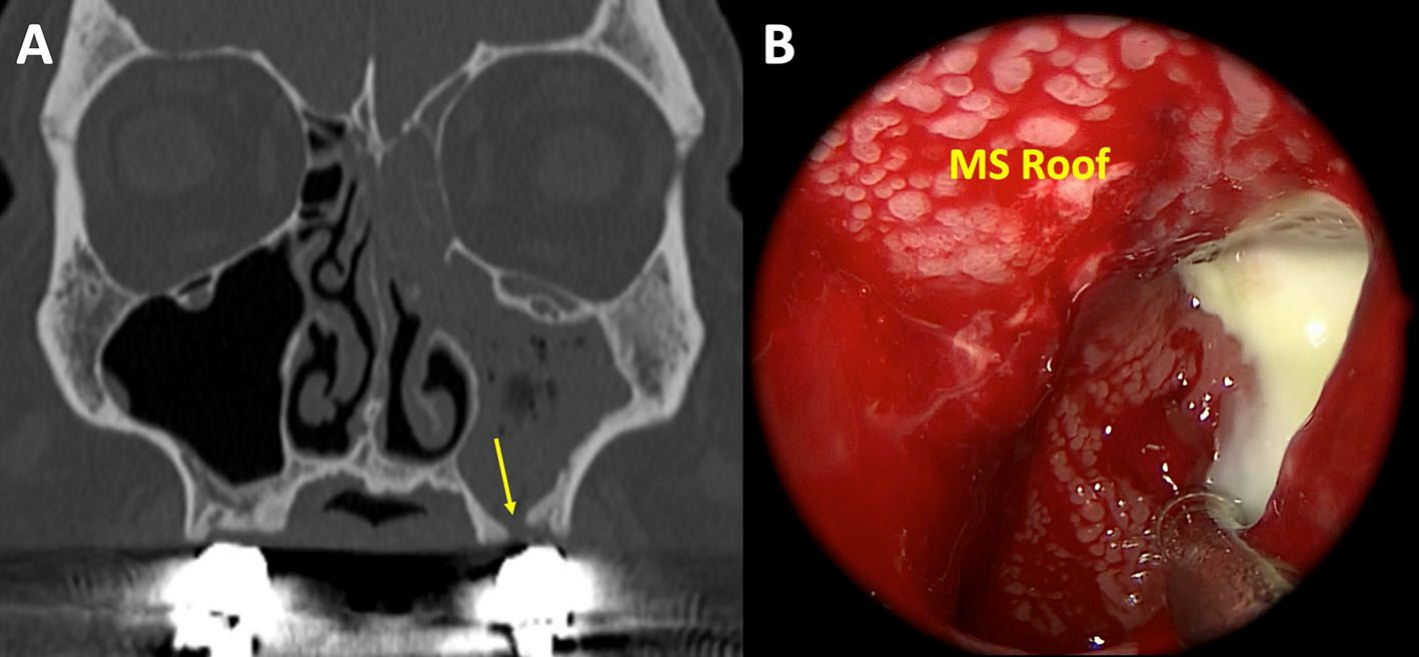
Characteristic example of odontogenic sinusitis. (A) Computed tomography showing left‐sided maxillary and ethmoid sinus opacification with bony dehiscence of the maxillary sinus (MS) floor after prior molar extraction and bridge placement (yellow arrow). (B) 70° nasal endoscopic view of the left MS lumen after wide maxillary antrostomy, demonstrating characteristic diffuse papillary edema of all the MS walls.

**FIGURE 3:**
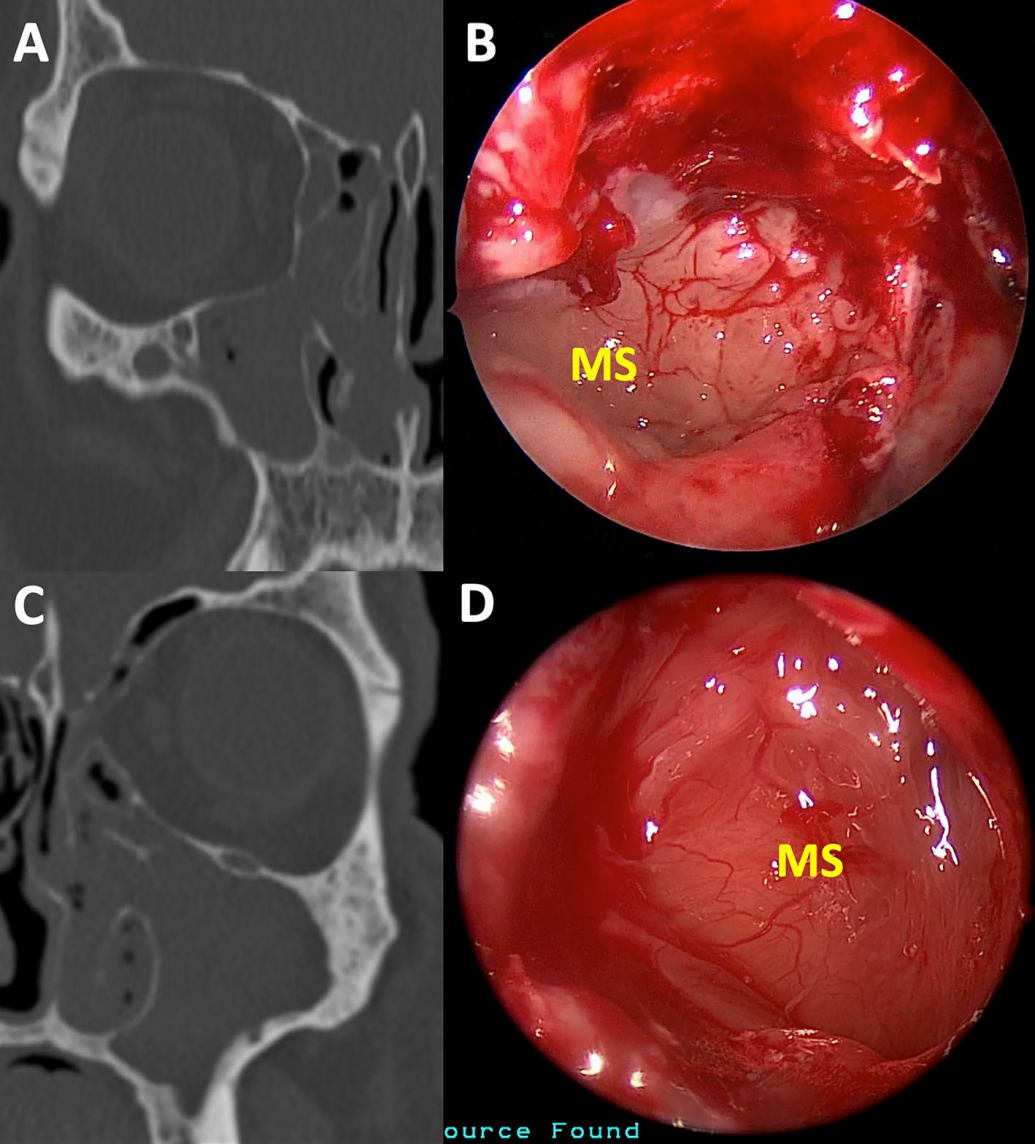
Examples of unilateral non‐odontogenic rhinosinusitis cases without maxillary sinus (MS) papillary edema. (A) Computed tomography (CT) showing right‐sided near‐complete opacification of the maxillary and anterior ethmoid sinuses in the setting of chronic rhinosinusitis with nasal polyps. (B) 70° nasal endoscopic view of the right MS lumen after maxillary antrostomy in the patient from (A). While edema was present, there were no papillary changes to the mucosal edema. (C) CT showing left‐sided maxillary and ethmoid sinus opacification in the setting of purulent non‐odontogenic rhinosinusitis without nasal polyps. (D) 70° nasal endoscopic view of the left MS lumen after modified endoscopic medial maxillectomy in the patient from (C). While the sinus walls were edematous, no papillary surface changes were seen.

**FIGURE 4:**
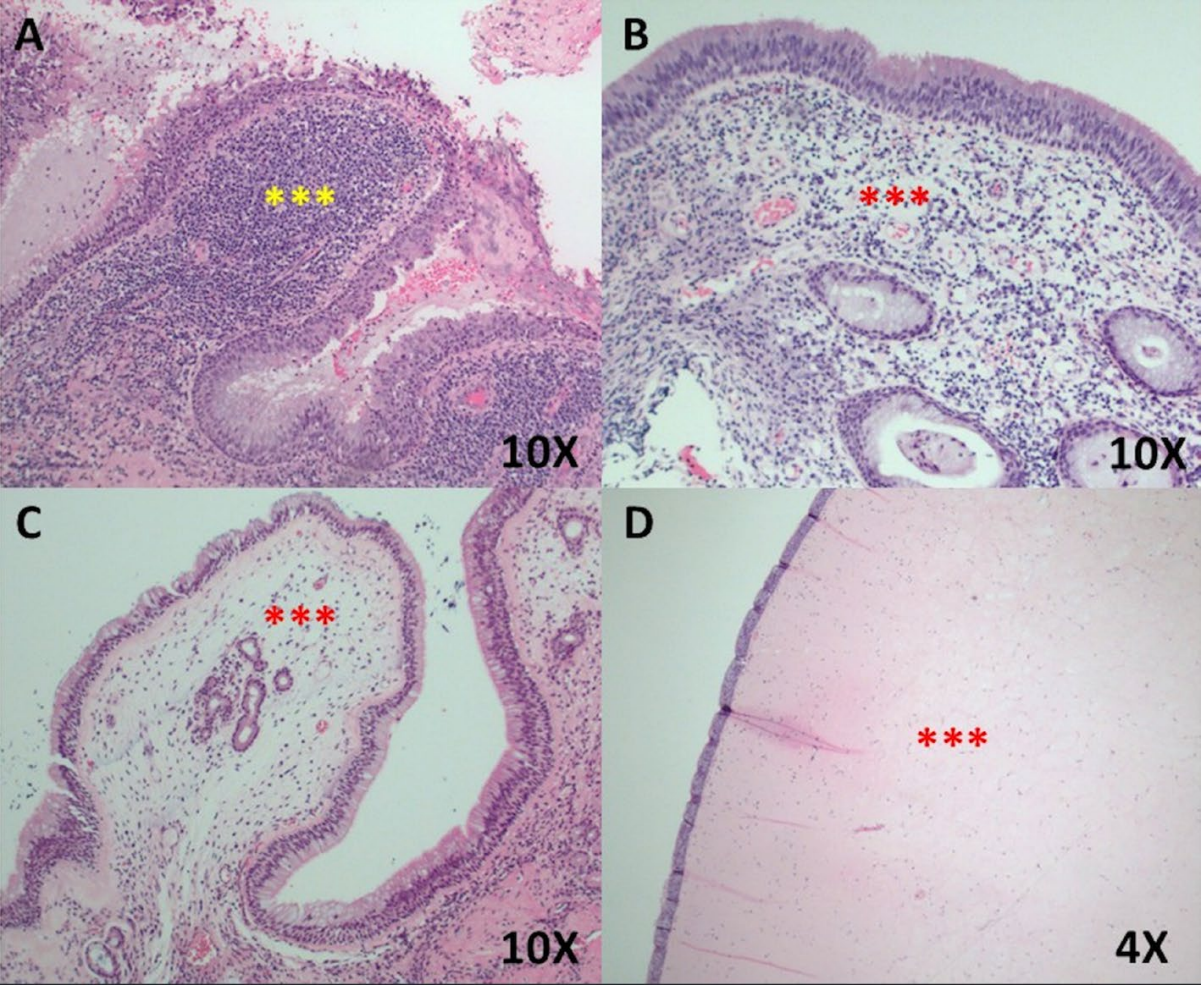
Representative histologic images in the different study cohorts. (A) Odontogenic sinusitis with maxillary sinus papillary edema (MSPE), ×10. The mucosal papillary protrusion demonstrated a dense subepithelial lymphocytic infiltration (triple yellow asterisk). (B) Chronic rhinosinusitis without nasal polyps (CRSsNP) without MSPE, ×10. An intact pseudostratified columnar epithelium was seen overlying subepithelial edema and a predominantly lymphocytic infiltrate (triple red asterisk). (C) CRSsNP but with MSPE, ×10. The mucosal papillary protrusion demonstrated subepithelial edema, but significantly less dense inflammatory infiltration (triple yellow asterisk) compared to that seen in (A). (D) Chronic rhinosinusitis with nasal polyps, without MSPE, ×4. Significant subepithelial edema was demonstrated with minimal inflammatory cell infiltration (triple red asterisk).

Figures 2, 3, and S1 show representative examples of different cases that underwent blinded endoscopic image review. The inter‐rater agreement analysis revealed almost perfect agreement with a Fleiss *κ* score of 0.927, with a 95% confidence interval of (0.858, 0.997) (*p* < 0.0001). Tables 2, 3, 4 show pairwise comparisons between each cohort pertaining to the presence of maxillary sinus edema, MSPE, and disease extents in each paranasal sinus on CT. Table 2 shows that for ODS versus infectious CRSsNP, MSPE was significantly more likely in ODS (100% vs. 22.7%, *p* < 0.0001). Additionally, ODS patients demonstrated significantly greater frequencies of maxillary, anterior ethmoid, and frontal sinus involvement on CT. Table 3 shows that for ODS versus noninfectious CRSwNP, MSPE (100% vs. 0%, *p* < 0.0001) and maxillary sinus opacification (100% vs. 61.5%, *p* = 0.0001) were significantly more likely in ODS, while posterior ethmoid and sphenoid sinus opacification were more likely in CRSwNP (*p* ≤ 0.0001). Table 4 shows that for infectious CRSsNP versus noninfectious CRSwNP, opacification of anterior and posterior ethmoid, frontal, and sphenoid sinuses on CT was significantly more likely in CRSwNP compared to infectious CRSsNP (*p* ≤ 0.006). Rates of MSPE were not significantly different between the CRSsNP and CRSwNP groups (22.7% vs. 0%, *p* = 0.160). A predictive model for MSPE demonstrated 100% sensitivity, 89.1% specificity, 85.7% PPV, and 100% NPV in predicting ODS (Table S1). Figure 4 shows characteristic histologic features patients with ODS, CRSsNP with and without MSPE, and CRSwNP.

**TABLE 2:** Comparison of maxillary sinus edema, papillary edema (MSPE), and extent of disease in different paranasal sinuses on computed tomography (CT) between patients with odontogenic sinusitis and infectious chronic rhinosinusitis without nasal polyps (CRSsNP).

Variables	ODS (*n* = 41)	Infectious CRSsNP (*n* = 22)	*p*
Any maxillary sinus edema, [*n* (%)]	40 (100.0)	22 (100.0)	1.000
Maxillary sinus papillary edema, [*n*, (%)]	41 (100.0)	5 (22.7)	**< 0.0001**
Maxillary sinus disease on CT [*n*, (%)]			
None or MT	0 (0.0)	6 (27.3)	**0.001**
Partial to complete opacification	41 (100.0)	16 (72.7)	
Anterior ethmoid sinus disease on CT [*n*, (%)]			
None or MT	7 (17.1)	19 (86.4)	**< 0.0001**
Partial to complete opacification	34 (82.9)	3 (13.6)	
Posterior ethmoid sinus disease on CT [*n*, (%)]			
None or MT	40 (97.6)	21 (95.5)	0.894
Partial to complete opacification	1 (2.4)	1 (4.5)	
Sphenoid sinus disease on CT [*n*, (%)]			
None or MT	41 (100.0)	22 (100.0)	1.000
Partial to complete opacification	0 (0.0)	0 (0.0)	
Frontal sinus disease on CT [*n*, (%)]			
None or MT	27 (65.9)	22 (100.0)	**0.006**
Partial to complete opacification	14 (34.1)	0 (0.0)	

*Note*: Bolded *p* values represent statistical significance.

Abbreviation: MT, mucosal thickening.

**TABLE 3:** Comparison of maxillary sinus edema, papillary edema (MSPE), and extent of disease in different paranasal sinuses on computed tomography (CT) between patients with odontogenic sinusitis and noninfectious chronic rhinosinusitis with nasal polyps (CRSwNP).

	ODS (*n* = 41)	Noninfectious CRSwNP (*n* = 13)	*p*
Any maxillary sinus edema, [*n* (%)]	40 (100.0)	12 (92.3)	0.185
Maxillary sinus papillary edema, [*n*, (%)]	41 (100.0)	0 (0.0)	**< 0.0001**
Maxillary sinus disease on CT [*n*, (%)]			
None or MT	0 (0.0)	5 (38.5)	**0.0001**
Partial to complete opacification	41 (100.0)	8 (61.5)	
Anterior ethmoid sinus disease on CT [*n*, (%)]			
None or MT	7 (17.1)	2 (15.4)	0.989
Partial to complete opacification	34 (82.9)	11 (84.6)	
Posterior ethmoid sinus disease on CT [*n*, (%)]			
None or MT	40 (97.6)	6 (46.2)	**< 0.0001**
Partial to complete opacification	1 (2.4)	7 (53.8)	
Sphenoid sinus disease on CT [*n*, (%)]			
None or MT	41 (100.0)	8 (61.5)	**0.0001**
Partial to complete opacification	0 (0.0)	5 (38.5)	
Frontal sinus disease on CT [*n*, (%)]			
None or MT	27 (65.9)	8 (61.5)	0.957
Partial to complete opacification	14 (34.1)	5 (38.5)	

*Note*: Bolded *p* values represent statistical significance.

Abbreviation: MT, mucosal thickening.

**TABLE 4:** Comparison of maxillary sinus edema, papillary edema (MSPE), and extent of disease in different paranasal sinuses on computed tomography (CT) between patients with infectious chronic rhinosinusitis without nasal polyps (CRSsNP) and noninfectious chronic rhinosinusitis with nasal polyps (CRSwNP).

	Infectious CRSsNP (*n* = 22)	Noninfectious CRSwNP (*n* = 13)	*p*
Any maxillary sinus edema [*n* (%)]	22 (100.0)	12 (92.3)	0.395
Maxillary sinus papillary edema [*n*, (%)]	5 (22.7)	1 (7.7)	0.499
Maxillary sinus disease on CT [*n*, (%)]			
None or MT	6 (27.3)	5 (38.5)	0.776
Partial to complete opacification	16 (72.7)	8 (61.5)	
Anterior ethmoid sinus disease on CT [*n*, (%)]			
None or MT	19 (86.4)	2 (15.4)	**0.0001**
Partial to complete opacification	3 (13.6)	11 (84.6)	
Posterior ethmoid sinus disease on CT [*n*, (%)]			
None or MT	21 (95.5)	6 (46.2)	**0.003**
Partial to complete opacification	1 (4.5)	7 (53.8)	
Sphenoid sinus disease on CT [*n*, (%)]			
None or MT	22 (100.0)	8 (61.5)	**0.006**
Partial to complete opacification	0 (0.0)	5 (38.5)	
Frontal sinus disease on CT [*n*, (%)]			
None or MT	22 (100.0)	8 (61.5)	**0.006**
Partial to complete opacification	0 (0.0)	5 (38.5)	

*Note*: Bolded *p* values represent statistical significance.

Abbreviation: MT, mucosal thickening.

Table S2 shows bacterial culture results from ODS and infectious CRSsNP patients. Overall, oral and anaerobic species were significantly more common in ODS compared to CRSsNP patients. Oral bacteria were identified in 37/41 (90.2%) ODS patients versus 3/22 (13.6%) CRSsNP patients (*p* < 0.0001), and anaerobes were found in 29/41 (70.7%) ODS patients versus 1/22 (4.5%) CRSsNP patients (*p* < 0.0001). Bacterial species that were significantly more likely in ODS were oral streptococcal species and non‐speciated anaerobic flora. Bacterial species that were significantly more likely in infectious CRSsNP were *Pseudomonas aeruginosa, Staphylococcus aureus* (methicillin‐sensitive and resistant), and *Escherichia coli* . Of note, in the three infectious CRSsNP patients who grew ODS‐related bacteria on cultures, none demonstrated MSPE.

## 4 Discussion

ODS is a purulent sinusitis stemming from infectious dental sources, and is the most common cause of unilateral maxillary sinusitis [4]. The dental and sinus infections lead to an innate and Th1 or Th17‐driven severe inflammatory sinus mucosal edema that is predominantly lymphocytic [13, 16]. ODS patients most commonly also maintain a normal pseudostratified columnar epithelial lining [13, 14]. The sinus inflammatory reaction in ODS then commonly leads to the characteristic papillary appearance of the edematous mucosa, or MSPE.

Zhang et al. published the first study on MSPE in ODS. They reported papillary mucosal protrusions in 25 ODS patients based on H&E staining of MS mucosal specimens. While they made multiple comparisons between their ODS, CRSsNP (*n* = 7), and CRSwNP (*n* = 10) cohorts with regard to inflammatory infiltrates on H&E, claudin‐4 tight junction protein staining, and cytokine profiles, they did not specifically compare the presence of MSPE on endoscopy or H&E between their patient cohorts [13]. Sato et al. subsequently showed in 20 ODS patients that all had both maxillary sinus purulence and MSPE on nasal endoscopy, and mucosal papillary protrusions on H&E staining [14]. While these studies showed that MSPE was common in ODS patients, they did not compare the presence of MSPE to those with non‐odontogenic sinus disease.

The current study built upon these prior works, and showed via blinded endoscopic review that MSPE was more common in ODS than both infectious CRSsNP and noninfectious CRSwNP, demonstrating 100% sensitivity and 89% specificity. Also encouraging was the almost perfect agreement between three blinded rhinologists when assessing for MSPE, implying that MSPE is a reliable endoscopic finding that surgeons can readily identify. While further research is necessary, these findings could prove beneficial not only for diagnosing patients with USD, but also in furthering the understanding of certain aspects of sinusitis pathophysiology and therapeutic prognosis.

Regarding diagnostic implications, MSPE could have an emerging role in the evaluation of USD, providing an additional clue of an odontogenic source. Despite many ODS patients having a clear history of maxillary dental disease or treatments, and nasal endoscopy or CT demonstrating adjacent purulent maxillary sinusitis, ODS can still evade clinicians for a few reasons. First, up to 30% of patients will have subtle to absent overt dental pathology on CT imaging due to either absent dentition, or early stage dental infections that show minimal to no bone erosion around the tooth roots [1, 2, 9, 11]. Second, even if there is overt maxillary dental pathology on CT, radiologists overlook the dental conditions in the majority of cases [3, 11, 17, 18], so detection often depends on otolaryngologists. ODS patients also rarely complain of dental pain, and may not divulge their dental history [2, 19]. Another issue is that while ODS patients are more likely to have oral and anaerobic organisms grow on sinus cultures [10], some patients' cultures will show no growth or fail to speciate adequately. Lastly, while certain clinical features increase the likelihood of ODS (e.g., unilateral middle meatal purulence on nasal endoscopy, subjective foul‐smelling drainage, and CT demonstrating relative sparing of the posterior ethmoid and sphenoid sinuses) [9], these features are not always be present. Based on the high sensitivity and specificity of MSPE for ODS, the current study showed that MSPE can help with suspecting an odontogenic cause of USD, especially if the previously mentioned ODS predictive features are not present. As a corollary, with 100% NPV, the absence of MSPE in USD patients may help surgeons rule out ODS. Taken a step further, future studies should also consider whether mucosal histologic features help discern ODS from other sinusitis phenotypes. Ultimately, the aim of improving ODS suspicion and recognition is to ensure referrals to dental specialists for optimal dental evaluations and management.

Another important point to discuss centers on when to use MSPE to facilitate ODS suspicion. Since only MSPE was assessed, this study would technically only support MSPE identification as being helpful to predict ODS during or after maxillary antrostomy. While this would still be helpful to surgeons who performed ESS without preoperative ODS suspicion, the utility of identifying extramaxillary papillary edema in predicting ODS before maxillary antrostomy would be even greater, albeit speculative. That said, identifying extramaxillary papillary edema could still facilitate ODS suspicion. For example, Figure S2 highlights two ODS patients with middle meatal purulence and papillary edema. If papillary edema is seen in infected sinonasal cavities, surgeons should still consider a possible odontogenic source, and study CT scans for possible overt dental pathology.

Considering MSPE as a distinct mucosal inflammatory response could also improve the pathophysiologic understanding of all forms of rhinosinusitis, which might have therapeutic prognostic implications as well. In this study, MSPE only occurred in patients with purulent maxillary sinusitis, whether odontogenic or non‐odontogenic. While the reasons for MSPE being more common in ODS are speculative, multiple explanations could be postulated. First, the sinus mucosa may mount a unique inflammatory response to odontogenic organisms not typically encountered in the sinus lumen. While there were significant differences in oral and anaerobic bacteria between the ODS and infectious CRSsNP patients in this study, whether bacterial species differed in ODS and CRSsNP patients with MSPE could not be assessed statistically due to the small sample size of MSPE in CRSsNP (5/22). Interestingly, while 3/22 CRSsNP patients harbored possible odontogenic organisms, none of these patients had MSPE. While this could suggest that oral bacteria are not the sole driver of MSPE development, larger studies would be needed to determine whether specific bacteria cause MSPE. Also important to note, while MSPE was significantly less common in CRSsNP than ODS, the 23% rate of MSPE in infectious CRSsNP should not be disregarded. The only study to date to compare ODS to CRS patients histologically was by Zhang et al., who showed that ODS patients' epithelial tight junctions were stronger than CRS patients based on claudin‐4 staining intensity [13]. However, they did not report whether their CRS patients demonstrated MSPE. Rather than MSPE being specific to ODS and oral organisms, perhaps MSPE instead signifies a more adaptive mucosal immune response to intraluminal or intramucosal bacteria, regardless of bacterial origin. For example, as suggested by Zhang et al., MSPE may allow for increased antimicrobial action by increasing mucosal surface area via packing more papillary protrusions into the confined maxillary sinus space. This could allow for both enhanced mucociliary clearance and delivery of inflammatory cells and antimicrobial products to the mucosal surface. Presuming sinus outflow tracts are adequately patent, this enhanced mucosal inflammatory response could lead to higher sinusitis resolution rates. This has been indirectly evidenced by multiple ODS studies showing 90%–98% long‐term success following ESS plus appropriate dental treatment, without the need for long‐term maintenance therapies like anti‐inflammatory or antimicrobial medications [20, 21]. Future studies could explore whether sinus papillary edema seen in infectious CRSsNP portends a more favorable prognosis following ESS compared to infectious CRSsNP without sinus papillary edema. Other potential mechanisms behind sinus papillary edema development that require further study include local mucosal inflammatory responses, mucosal remodeling pathways, and interactions between microbial biofilms and the mucosa.

Multiple study limitations should also be discussed. First, while statistically powered, the study still included relatively small sample sizes of each cohort. While the data showed a clear trend with ODS harboring MSPE more often than CRS patients, it is possible that some ODS patients will not have MSPE, and more patients with CRSsNP or CRSwNP could demonstrate MSPE. More research is needed to understand the significance of MSPE in all types of sinus disease. Another issue with the sample sizes was the inability to analyze whether revision ESS state could have associations with MSPE. Only 5/22 infectious CRSsNP patients had MSPE, and all the ODS patients had primary ESS, so these samples prevented statistical comparisons. Another limitation was using the infectious CRSsNP cohort for comparisons, considering CRSsNP is comprised of multiple possible etiologies. For example, Stevens et al. showed that CRSsNP patients demonstrated significant heterogeneity with regard to T1, T2, and T17 mucosal inflammatory endotypes [22]. Studies are needed to stratify CRSsNP further into different phenotypes and endotypes, and determine whether certain CRS types are more likely to demonstrate MSPE. Another limitation relates to the study's grading of MSPE as being either present or absent. Future studies could consider grading MSPE extent based on certain features like different mucosal surface patterns seen, presence of polypoid outgrowths of the edematous mucosa, number of sinus walls involved, and extramaxillary sinus involvement. Such a grading system could further improve the diagnostic utility of sinus papillary edema. Another potential limitation was that different endoscopic image capture systems were used between patients. For example, some cases were recorded with white light endoscopy, and others with Storz Professional Image Enhancement System (SPIES) endoscopy. Prior studies have reported the utility of narrow band imaging and SPIES in detecting certain sinonasal pathologies based on mucosal surface and submucosal microvascular changes [23, 24]. Future studies could explore the utility of evaluating MSPE with these other imaging techniques. A final potential limitation was the lack of allergy testing across all patients. Whether allergic inflammation affects the MSPE formation cannot be determined.

## 5 Conclusions

MSPE is a distinct finding that is reliably identifiable, and is significantly more likely in ODS compared to infectious CRSsNP and noninfectious CRSwNP. With 100% sensitivity and NPV, MSPE absence makes ODS very unlikely. With 86% PPV, MSPE is not pathognomonic for ODS, but should arouse suspicion for a possible odontogenic source.

## Funding

The authors have nothing to report.

## Conflicts of Interest

Edward D. McCoul is a consultant for 3D Matrix, Advanced Rx, Scopi Medical, Zsquare; speaker for Sanofi/Regeneron. John R. Craig is a research consultant for Aerin Medical Inc. The other authors declare no conflicts of interest.

## Supporting information


**Figure S1:** Different appearances of maxillary sinus papillary edema (MSPE) on 70° nasal endoscopic viewing of right maxillary sinuses, in patients with odontogenic sinusitis (ODS) and infectious non‐odontogenic rhinosinusitis without nasal polyps. (A, B) Examples of MSPE in two ODS patients. In some cases, the papillary protrusions arose on larger polypoid outgrowths (A), while in other cases the MSPE was seen along flatter areas of edema (B). (C, D) Other examples of MSPE in two patients with infectious CRSsNP. In these examples, the papillary surface changes occurred along flatter areas of MS wall edema.

**Figure S2:** Examples of papillary edema being seen in two different odontogenic sinusitis (ODS) patients outside of the maxillary sinus (MS). (A) Left nasal endoscopic view of the anterior aspect of the middle meatus before any endoscopic sinus surgery (ESS) was performed. The uncinate process (UP) was bulged medially and demonstrated papillary edema, and this edema was also seen along the neighboring lateral nasal wall (LNW). (B) Another example of papillary edema before any ESS in a different ODS patient. Here the middle turbinate (MT) demonstrated papillary edema that was in contact with middle meatal purulence.

**Table S1:** Predictive model pertaining to the utility of maxillary sinus papillary edema in predicting odontogenic sinusitis compared to infectious and noninfectious forms of chronic rhinosinusitis. NPV, negative predictive value; PPV, positive predictive value.

**Table S2:** Bacterial species identified in maxillary sinus cultures in patients with odontogenic sinusitis (ODS) and infectious non‐odontogenic chronic rhinosinusitis without nasal polyps (CRSsNP). MRSA, methicillin‐resistant staphylococcus aureus; MSSA, methicillin‐sensitive staphylococcus aureus; spp, species. Bold *p* values show statistical significance.

## Data Availability

The data that support the findings of this study are available from the corresponding author upon reasonable request.
