# Supplementary figures and images for: Maxillary Sinus Papillary Edema as a Predictor of Odontogenic Sinusitis
Source: Laryngoscope. 2025 Dec 19;136(5):2082–91. doi: 10.1002/lary.70323 (PMC13067223; doi:10.1002/lary.70323)

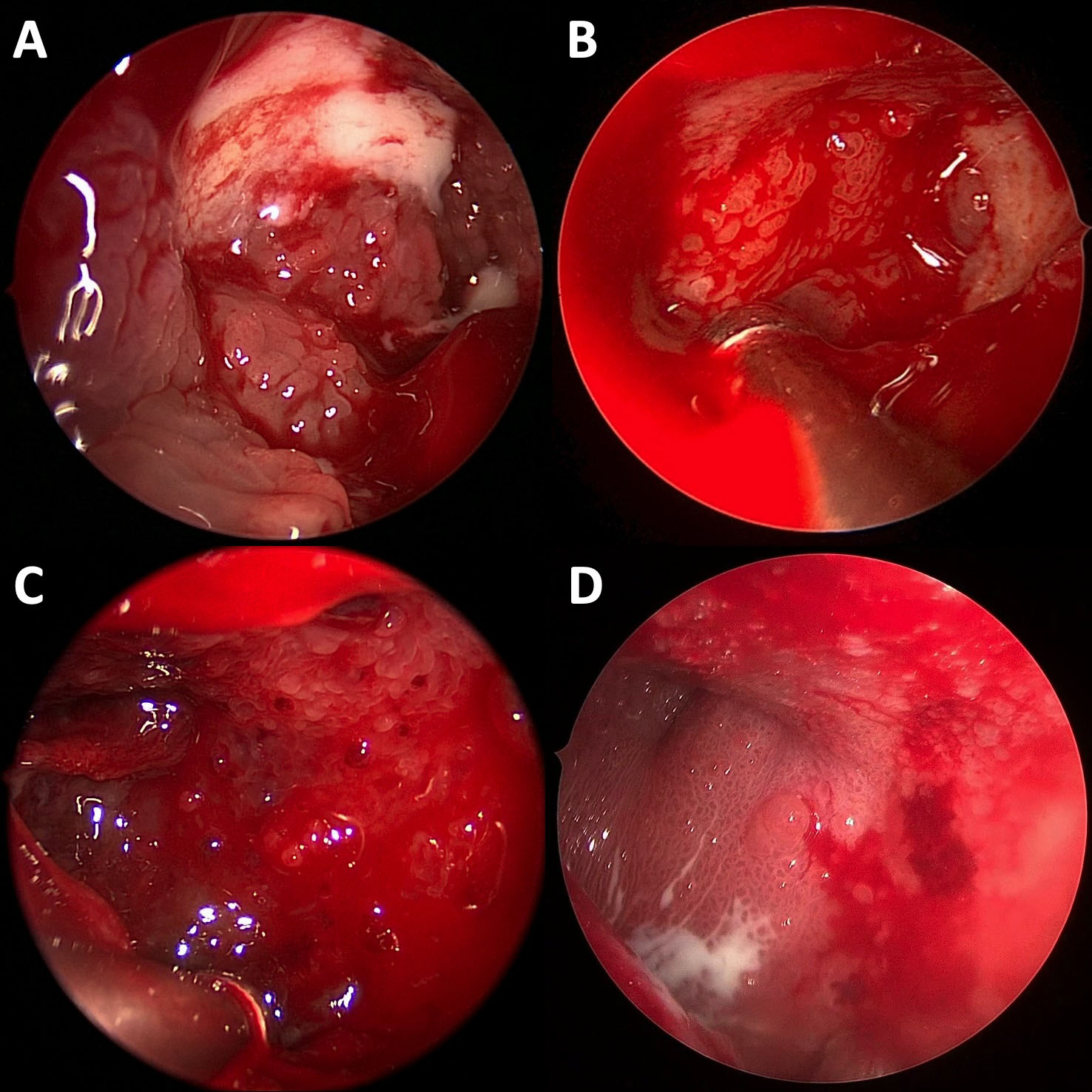

Supplement: Supplementary file 1 — Figure S1: Different appearances of maxillary sinus papillary edema (MSPE) on 70° nasal endoscopic viewing of right maxillary sinuses, in patients with odontogenic sinusitis (ODS) and infectious non‐odontogenic rhinosinusitis without nasal polyps. (A, B) Examples of MSPE in two ODS patients. In some cases, the papillary protrusions arose on larger polypoid outgrowths (A), while in other cases the MSPE was seen along flatter areas of edema (B). (C, D) Other examples of MSPE in two patients with infectious CRSsNP. In these examples, the papillary surface changes occurred along flatter areas of MS wall edema. [file LARY-136-2082-s002.jpg]

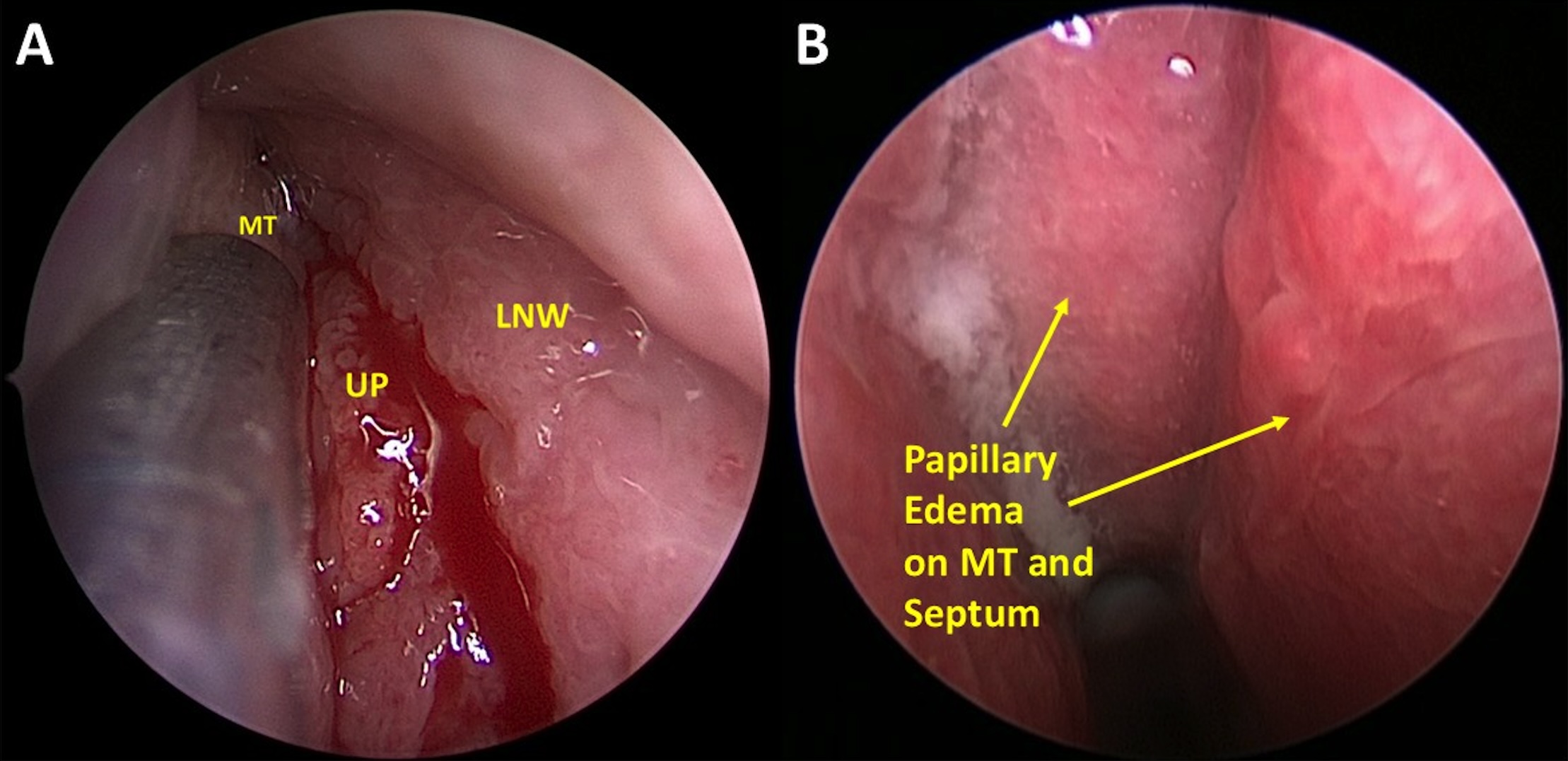

Supplement: Supplementary file 2 — Figure S2: Examples of papillary edema being seen in two different odontogenic sinusitis (ODS) patients outside of the maxillary sinus (MS). (A) Left nasal endoscopic view of the anterior aspect of the middle meatus before any endoscopic sinus surgery (ESS) was performed. The uncinate process (UP) was bulged medially and demonstrated papillary edema, and this edema was also seen along the neighboring lateral nasal wall (LNW). (B) Another example of papillary edema before any ESS in a different ODS patient. Here the middle turbinate (MT) demonstrated papillary edema that was in contact with middle meatal purulence. [file LARY-136-2082-s004.jpg]
