# Supplementary material for: Maxillary Sinus Papillary Edema as a Predictor of Odontogenic Sinusitis
Source: Laryngoscope. 2025 Dec 19;136(5):2082–91. doi: 10.1002/lary.70323 (PMC13067223; doi:10.1002/lary.70323)
Supplement: Supplementary file 3 — Table S1: Predictive model pertaining to the utility of maxillary sinus papillary edema in predicting odontogenic sinusitis compared to infectious and noninfectious forms of chronic rhinosinusitis. NPV, negative predictive value; PPV, positive predictive value. [file LARY-136-2082-s001.docx]

**Supplemental Table I**

| **Statistic** | **Value** | **Standard Error** | **95% Confidence Limits** | |
| --- | --- | --- | --- | --- |
| Sensitivity | 1.0000 | 0.0000 | 1.0000 | 1.0000 |
| Specificity | 0.8913 | 0.0459 | 0.8014 | 0.9813 |
| PPV | 0.8571 | 0.0591 | 0.7412 | 0.9731 |
| NPV | 1.0000 | 0.0000 | 1.0000 | 1.0000 |

Predictive model pertaining to the utility of maxillary sinus papillary edema in predicting odontogenic sinusitis compared to infectious and non-infectious forms of chronic rhinosinusitis. PPV, positive predictive value; NPV, negative predictive value.
