# Supplementary material for: Maxillary Sinus Papillary Edema as a Predictor of Odontogenic Sinusitis
Source: Laryngoscope. 2025 Dec 19;136(5):2082–91. doi: 10.1002/lary.70323 (PMC13067223; doi:10.1002/lary.70323)
Supplement: Supplementary file 4 — Table S2: Bacterial species identified in maxillary sinus cultures in patients with odontogenic sinusitis (ODS) and infectious non‐odontogenic chronic rhinosinusitis without nasal polyps (CRSsNP). MRSA, methicillin‐resistant staphylococcus aureus; MSSA, methicillin‐sensitive staphylococcus aureus; spp, species. Bold p values show statistical significance. [file LARY-136-2082-s003.docx]

**Supplemental Table II**

| **Bacteria** | **Infectious CRSsNP**  **(n=22)** | **ODS**  **(n=41)** | **p-value** |
| --- | --- | --- | --- |
| **None** | 0 (0.0%) | 2 (4.9%) | 0.538 |
| **Oral** | 3 (13.6%) | 37 (90.2%) | **<0.0001** |
| Oral streptococcal spp. | 2 (9.1%) | 22 (53.7%) | **0.0004** |
| *Streptococcus intermedius* | 0 (0.0%) | 8 (19.5%) | **0.042** |
| *Streptococcus constellatus* | 0 (0.0%) | 8 (19.5%) | **0.042** |
| *Streptococcus anginosus* | 1 (4.5%) | 2 (4.9%) | 1.000 |
| Streptococcus Group F | 0 (0.0%) | 2 (4.9%) | 0.538 |
| *Streptococci microaerophillic* | 0 (0.0%) | 1 (2.4%) | 1.000 |
| *Streptococcus sanguinis* | 0 (0.0%) | 1 (2.4%) | 1.000 |
| *Streptoccocus mitis* | 0 (0.0%) | 1 (2.4%) | 1.000 |
| *Streptococcus parasanguinis* | 1 (4.5%) | 0 (0.0%) | 0.349 |
| Prevotella spp.* | 0 (0.0%) | 3 (7.3%) | 0.546 |
| *Prevotella denticola* | 0 (0.0%) | 1 (2.4) | 1.000 |
| *Prevotella oralis* | 0 (0.0%) | 1 (2.4%) | 1.000 |
| *Prevotella loescheii* | 0 (0.0%) | 1 (2.4%) | 1.000 |
| *Prevotella melaninogenica* | 0 (0.0%) | 1 (2.4%) | 1.000 |
| Other oral species | | | |
| *Fusobacterium* spp.* | 0 (0.0%) | 7 (17.1%) | 0.086 |
| *Eikenella* spp. | 0 (0.0%) | 4 (9.8%) | 0.288 |
| *Actinomyces** | 0 (0.0%) | 1 (2.4%) | 1.000 |
| *Micrococcus* | 0 (0.0%) | 1 (2.4%) | 1.000 |
| *Aggregatibacter aphrophilus* | 0 (0.0%) | 1 (2.4%) | 1.000 |
| *Peptostreptococcus micros** | 0 (0.0%) | 1 (2.4%) | 0.538 |
| *Peptostreptococcus magnus** | 0 (0.0%) | 1 (2.4%) | 1.000 |
| *Peptostreptococcus anaerobius** | 0 (0.0%) | 1 (2.4% | 1.000 |
| *Bacteroides fragilis** | 0 (0.0%) | 1 (2.4%) |  |
| **Anaerobes** | 1 (4.5%) | 29 (70.7%) | **<0.0001** |
| Other non-oral anaerobes | | | |
| Anaerobic flora (non-  speciated) | 1 (4.5%) | 16 (39.0%) | **0.003** |
| *Propionibacterium acnes* | 0 (0.0%) | 1 (2.4%) | 1.000 |
| **Aerobes, non-oral** |  |  |  |
| MSSA | 9 (40.9%) | 1 (2.4%) | **0.0002** |
| *Pseudomonas aeruginosa* | 8 (36.4%) | 0 (0.0%) | **<0.0001** |
| Coagulase-negative staphylococcus | 5 (22.7%) | 13 (31.7%) | 0.564 |
| MRSA | 4 (18.2%) | 0 (0.0%) | **0.012** |
| *Escherichia coli* | 3 (13.6%) | 0 (0.0%) | **0.039** |
| *Haemophilus influenza* | 3 (13.6%) | 1 (2.4%) | 0.118 |
| *Streptococcus pneumoniae* | 2 (9.1%) | 0 (0.0%) | 0.118 |
| *Neisseria* spp. | 1 (4.5%) | 2 (4.9%) | 1.0000 |
| *Enterobacter cloacae* | 1 (4.5%) | 0 (0.0%) | 0.349 |
| *Proteus mirabalis* | 1 (4.5%) | 0 (0.0%) | 0.349 |
| *Staphylococcus agalactiae* | 1 (4.5%) | 0 (0.0%) | 0.349 |
| *Staphylococcus epidermidis* | 0 (0.0%) | 3 (7.3%) | 0.546 |
| *Corynebacterium* spp. | 0 (0.0%) | 7 (17.1%) | 0.086 |
| *Staphylococcus lugdunensis* | 0 (0.0%) | 2 (4.9%) | 0.538 |
| *Moraxella nonliquefaciens* | 0 (0.0%) | 1 (2.4%) | 1.000 |
| *Klebsiella oxytoca* | 0 (0.0%) | 1 (2.4%) | 1.000 |

Bacterial species identified in maxillary sinus cultures in patients with odontogenic sinusitis (ODS) and infectious non-odontogenic chronic rhinosinusitis without nasal polyps (CRSsNP). Spp, species; MSSA, methicillin-sensitive staphylococcus aureus; MRSA, methicillin-resistant staphylococcus aureus. Bold p-values show statistical significance.
